# Changes in glycemic control from 1996 to 2006 among adults with type 2 diabetes: a longitudinal cohort study

**DOI:** 10.1186/1472-6963-10-158

**Published:** 2010-06-09

**Authors:** Karen J Blumenthal, Mary E Larkin, Gail Winning, David M Nathan, Richard W Grant

**Affiliations:** 1 The Diabetes Center, Massachusetts General Hospital, 50 Staniford Street, Boston MA, 02114, USA; 2 Division of General Medicine, Massachusetts General Hospital, Fruit Street, Boston MA, 02114, USA; 3 Harvard Medical School, Shattuck Street, Boston, MA 02115, USA

## Abstract

**Background:**

Our objectives were to examine temporal changes in HbA1c and lipid levels over a 10-year period and to identify predictors of metabolic control in a longitudinal patient cohort.

**Methods:**

We identified all adults within our hospital network with T2DM who had HbA1c's measured in both 1996 and 2006 (longitudinal cohort). For patients with no data in 2006, we used hospital and social security records to distinguish patients lost to follow-up from those who died after 1996. We compared characteristics of the 3 baseline cohorts (longitudinal, lost to f/u, died) and examined metabolic trends in the longitudinal cohort.

**Results:**

Of the 4944 patients with HbA1c measured in 1996, 1772 (36%) had an HbA1c measured in 2006, 1296 (26%) were lost to follow-up, and 1876 (38%) had died by 2006. In the longitudinal cohort, mean HbA1c decreased by 0.4 ± 1.8% over the ten-year span (from 8.2% ± 1.7% to 7.8% ± 1.4%) and mean total cholesterol decreased by 49.3 (± 46.5) mg/dL. In a multivariate model, independent predictors of HbA1c decline included older age (OR 1.41 per decade, 95% CI: 1.3-1.6, p < 0.001), baseline HbA1c (OR 2.9 per 1% increment, 2.6 - 3.2, p < 0.001), and speaking English (OR 2.1, 1.4-3.1, p < 0.001).

**Conclusions:**

Despite having had diabetes for an additional 10 years, patients in our longitudinal cohort had better glycemic and cholesterol control in 2006 than 1996. Greatest improvements occurred in patients with the highest levels in the baseline year.

## Background

As the incidence of diabetes continues to increase in the United States, it has become increasingly important to understand trends in progression of the disease over time [1]. Type 2 diabetes (T2D) is seen as an inexorably progressive disease, with beta cell failure and increasing peripheral insulin resistance leading to worsening glycemic control, microvascular complications such as retinopathy and peripheral neuropathy, and significant macrovascular morbidity [2-4]. Medical therapy, when applied effectively, can prevent or delay the development of long-term complications, mediated largely through improved control of blood pressure, glycemia, and lipids [3,5,6].

Several cross-sectional US national surveys have provided important population-level data on diabetes prevalence and metabolic control over time. Data from the National Health and Nutritional Examination Survey (NHANES), for example, have confirmed an increasing diabetes prevalence, increasing obesity among patients with diabetes, and an apparent trend towards improved metabolic control after years of worsening [7-10]. However, interpretation of these trends is confounded by changes in US demographics and changes in screening criteria over the past decade. One study from the National Health Interview Survey, for example, found that despite significant increases in the diabetes population total each year, the mean age for patients with diabetes remained constant over an 8 year period [11].

Longitudinal studies of defined patient populations, conversely, allow the identification of actual trends in metabolic control over time and can identify individual-level predictors of worsening or improved glycemic control over time. We used records from a large hospital network to examine trends in both glycemic and lipid control over a 10-year span. We sought to determine whether changes in care practices would outweigh the impact of ten years of disease progression on glycemic and lipid control in a "real-world" cohort of patients with type 2 diabetes.

## Methods

### Setting and Patients

We used an electronic clinical data query to identify all adults with type 2 diabetes (T2D) receiving outpatient care at one of the 12 outpatient practices affiliated with Massachusetts General Hospital (MGH) in 1996. T2D was defined as 2 outpatient encounters or 1 inpatient encounter with T2D billing code (250.xx) during 1996 or an HbA1c > 7% prior to 1996. To further refine our T2D population, we excluded patients < 35 years of age and patients with "Type 1" modifiers in their electronic medical record (EMR) problem lists or ICD-9 billing codes.

We grouped the 4944 eligible patients into 3 cohorts: 1) the longitudinal cohort (patients who had at least one HbA1c assay performed in both 1996 and 2006); 2) patients who died before 2006, and 3) the lost to follow up cohort (the remaining patients with no 2006 data and no record of death). The study was approved by the Massachusetts General Hospital/Partners Health Care System Institutional Review Board.

### Clinical Variables

We used a combination of billing, laboratory, EMR, and appointment data sources to collect the following demographic and clinical data: age, gender, race, insurance status, primary language, HbA1c results and number of tests, number of outpatient visits, lipid results, and creatinine results. Vital status was determined using both hospital records and Social Security Administration's Death Master File [12].

Because the electronically-available data available for the overall cohort did not include the more detailed clinical information available from physician narrative progress notes in 1996, we also conducted two structured chart review analyses to address two secondary questions: 1) Among patients with very poor glycemic control in 1996 (HbA1c ≥ 11.5%, n = 152), did patients in the longitudinal cohort (n = 69) differ significantly from patients who were lost to follow-up (n = 83) in terms of medical and psychiatric comorbidity, substance abuse, or primary language spoken? And, 2) Among patients with moderately poor glycemic control in 1996, were patients who improved over the 10-year period more likely to receive insulin than patients who worsened? To address this second question, we conducted a 1:1 case-control analysis among the longitudinal cohort of patients with HbA1c levels between 8.0 and 9.0% in 1996 (n = 394) by randomly selecting 50 patients whose HbA1c was at least 1% lower in 2006 (cases) and 50 patients whose HbA1c was at least 1% higher in 2006 (controls). These patients were matched by age and gender.

The HbA1c results were obtained using a high-performance liquid chromatography method that has been described previously [9,13,14]. This assay serves as one of the primary reference laboratory methods for the National Glycohemoglobin Standardization Program and has inter- and intra-assay coefficients of variation <2.5%. Long-term drift is prevented by the use of long-term controls. Total plasma cholesterol and plasma triglyceride levels were measured enzymatically [15], high-density lipoprotein fraction was measured after precipitation of low-density, and very low-density lipoproteins were measured using dextran sulfate-magnesium [16]. We estimated LDL cholesterol levels indirectly using the Friedewald formula for patients with plasma triglyceride levels <400 mg/dL [17].

### Statistical Analysis

We compared the baseline characteristics of the 3 cohorts using analysis of variance and t-tests for continuous data and chi-square tests for categorical variables. For the longitudinal cohort, we examined individual trends in HbA1c and lipid control between 1996 and 2006. We separately modeled predictors of worsened glycemic control and worsened cholesterol control using multivariate regression modeling after assessing correlations between predictor variables. For the two chart review analyses, we used chi-square tests to compare proportions and report odds ratios. SAS (SAS v 9.1, Cary, North Carolina) was used for all analyses.

## Results

### Patient Characteristics

Of the 4944 eligible patients, 1772 (35.8%) had HbA1c assays performed at MGH in both 1996 and 2006; 1296 (26.2%) were lost to follow-up by 2006; and 1876 (37.9%) died (Table 1). The mean age for all eligible patients in 1996 was 60.0 (± 16.9) years, 47.9% were women, 23.4% were non-white, and 12.3% were non-English speaking. The mean HbA1c in 1996 was 8.3% (± 1.8).

**Table 1:** Patient Characteristics in 1996 by cohort status (n = 4944).

	All Patients	Characteristics by cohort			
	(n = 4944)	2006 Cohort (n = 1772, 35.8%)	Lost to F/U (n = 1296 26.2%)	Died (n = 1876, 37.9%)	**P-value**^**†**^
Age, years (SD)	60.0 (16.9)	58.8 (12.4)	59.7 (11.5)	70.6 (10.7)	<0.001
Women	47.9%	49.7%	50.2%	44.5%	0. 001
Non-White race/ethnicity	23.4%	21.7%	35.7%	16.4%	<0.001
Non-English Language	12.3%	10.2%	18.3%	10.2%	<0.001
Mean HbA1c % (SD)	8.3 (1.8)	8.2 (1.7)	8.4 (1.9)	8.3 (1.8)	0.008
Mean # of HbA1c tests (SD)	2.0 (1.1)	2.1 (1.1)	1.8 (1.1)	1.9 (1.1)	<0.001
Mean # of encounters (SD)	13.5 (11.8)	12.2 (10.7)	11.0 (10.4)	16.4 (13.9)	<0.001

"2006 cohort" = patients with HbA1c results in both 1996 and 2006; "Lost to F/U" = patients who were lost to follow-up prior to 2006; "Died" = patients who died prior to 2006; SD = Standard Deviation

^† ^P-values compare 2006 cohort (n = 1772) to lost-to-follow up cohort (n = 1549) to Died cohort (n = 1902)

Mean HbA1c and number of tests and outpatient encounters were those during the calendar year 1996.

The three cohorts varied significantly at baseline by age, sex, race/ethnicity, language, and HbA1c levels (Table 1). Patients who died in the intervening decade were more than a decade older (mean age 70.6 ± 10.75 years) than the other 2 cohorts (p < 0.001 for either comparison). Compared to the longitudinal cohort, the lost to follow-up cohort had a slightly higher mean HbA1c (8.4 ± 1.9% vs. 8.2 ± 1.7%, p = 0.002) and fewer HbA1c tests (1.8 ± 1.1 vs. 2.1 ± 1.1, p < 0.001) in 1996.

### Glycemic Control in the Longitudinal Cohort

**Figure 1:**
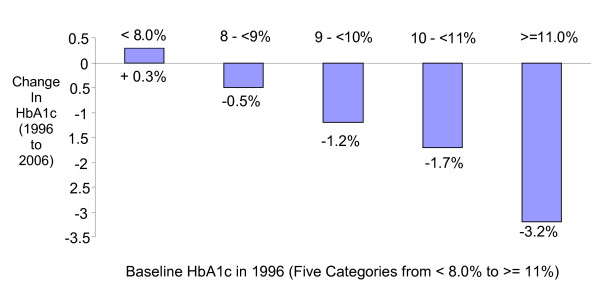
**Mean change in HbA1c between 1996 and 2006, by HbA1c level in 1996 (n = 1772)**.

The mean HbA1c decreased by 0.4 ± 1.8% (from 8.2% ± 1.7% to 7.8% ± 1.4% between 1996 and 2006) among patients in the longitudinal cohort with the majority (58.6%) having improved their glycemic control over this 10-year period. The group with the poorest glycemic control in 1996 experienced the biggest decreases in their HbA1c over the 10 years, while the patients with relatively good glycemic control in 1996 (HbA1c <= 8%) tended to have increased HbA1c by 2006 (Figure 1). The proportion of patients with HbA1c < 7.0% increased from 23.4% to 26.9% over the ten-year span (p = 0.02).

Using logistic regression, we found that higher baseline age (OR 1.41 per decade, 95% CI: 1.3-1.6, p < 0.001), higher baseline HbA1c (OR 2.9 per 1% higher HbA1c, 2.6 - 3.2, p < 0.001), and speaking English (OR 2.1, 1.4-3.1, p < 0.001) each independently predicted HbA1c improvement over the next 10 years (Table 2), while race, gender, creatinine and lipid levels, testing and visit frequency did not. A linear regression model with just these three covariates explained 51% of the variability in HbA1c change over time (adjusted R-squared 0.51).

**Table 2:** Baseline predictors of improved HbA1c or total cholesterol levels from 1996-2006, multivariate logistic regression models.

	HbA1c (n = 1772)	P-Value	Total Cholesterol (n = 1172)	P-Value
Age (decade)	1.41 (1.3-1.6)	<0.001	1.6 (1.3-1.9)	<0.001
Male gender	1.1 (1.6 to 3.6)	0.41	2.4 (1.6 - 3.6)	<0.001
English Speaking	2.1 (1.4-3.1)	0.01	1.0 (0.5 to 2.2)	0.95
1996 level	2.9 (2.6 - 3.2) (for HbA1c)	<0.001	1.04 (1.03-1.04) (for total cholesterol)	<0.001

This table presents the adjusted odds ratios (aOR) and 95% confidence intervals for two separate models; the HbA1c model identifies predictors of HbA1c improving from 1996 to 2006 after adjusting for all of the other variables; the Total cholesterol model identifies independent predictors of total cholesterol improving from 1996 to 2006 among patients with tests in both years (n = 1172), adjusted for the other variables.

**Figure 2:**
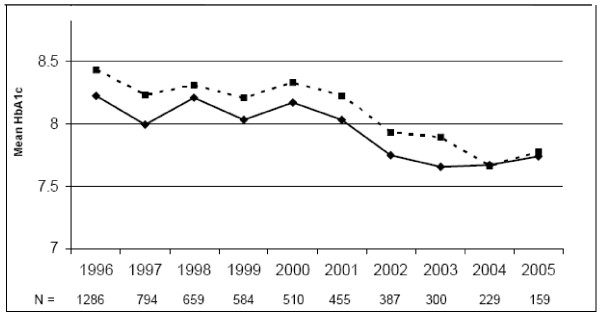
**Annual mean HbA1c from 1996-2005, comparing longitudinal (solid line, n = 1772 in all years) vs. lost to follow-up (dashed line, number if remaining patients for each year provided below graph)**.

Figure 2 presents the mean HbA1c per year from 1996 to 2005 for the longitudinal group. This figure also shows mean annual HbA1c levels for the lost to follow-up cohort for as many years as available for each patient. Although baseline HbA1c remained slightly higher from baseline onward in this second group, the improvement trend follows a similar pattern as the longitudinal cohort.

### Dyslipidemia in the Longitudinal Cohort

Total cholesterol results in both 1996 and 2006 were available for 1131 patients (63.8%) in the longitudinal cohort. In this subset of patients, 89.1% of patients had a decrease in total cholesterol and the mean total cholesterol decreased by an average of 49.3 (± 46.5) mg/dL between 1996 and 2006. The prevalence of LDL testing increased from 61.5% in 1996 to 84.1% in 2006. Of the 945 patients with LDL measurements in both periods, mean levels declined 54.0 (± 37.7) mg/dL, while HDL levels among patients measured in both periods (n = 1131) increased by 11.6 (± 11.4) mg/dL. Independently significant baseline predictors of total cholesterol improvement over 10 years included: Older age (OR 1.6 per decade, 95%CI: 1.3-1.92, p < 0.001), male gender (OR 2.4, 1.6 - 3.6, p < 0.001), and baseline cholesterol level (OR 1.04 per mg/dL, 1.03-1.04, p < 0.001) (Table 2). In a linear regression model of cholesterol change, these 3 covariates explained 48% of the model variability (adjusted R-squared 0.48).

### Results of Structured Chart Review Analyses

Among patients with very poor glycemic control (HbA1c > 11.5%) at baseline, structured medical chart review revealed that patients lost to follow up (n = 83) were more likely to be drug (7.2% vs. 1.5%, p = 0.04) or alcohol users (12.1% vs. 2.9%, p = 0.02), have a language barrier (22.9% vs. 8.7%, p = 0.05), and have no insurance or Medicaid (40.3% vs. 26.2%, p = 0.08) compared to patients who remained in care through to 2006 (n = 69). There were no significant differences in BMI, location of diabetes care, or prevalence of prescribed glycemic, blood pressure, lipid, or psychiatric medications between these case and control patients (data not shown).

In the nested case-control study of patients in the longitudinal cohort with moderately poor control at baseline (HbA1c 8.0-9.0%) matched by age and gender, we found that patients whose HbA1c significantly improved (cases) were less likely to take insulin in 1996 (40.0% vs. 66.0%, p = 0.045), have private insurance or Medicare in 1996 (93.0% vs. 76.6%, p = 0.03), or attend a diabetes specialty clinic (26.5% vs. 46.0%, p = 0.04 in 1996; 34.0% vs. 52.0%, p = 0.06 in 2006) than those whose HbA1c significantly worsened (controls). The mean weight loss (2.4 lbs vs. 5.3 lbs, p = 0.6) and mean increase in number of prescribed medications (4.2 vs. 4.3, p = 0.8) were not significantly different between cases and controls.

## Discussion

The results of this study demonstrate that among patients receiving clinical care in the same setting, glycemic control improved significantly between 1996 and 2006 despite the aging of the cohort and the 10-year increase in duration of their diabetes. Improvement in lipid control was even more pronounced, likely a consequence of the greater efficacy and ease of pharmacologic treatment for dyslipidemia relative to hyperglycemia. These results support the conclusion that national trends towards improved diabetes-related risk factor control are not solely the result of "down-staging" due to greater screening and earlier diabetes diagnosis, but reflect significantly more effective disease control for individuals living with diabetes.

Three patient factors (older age, higher HbA1c, and speaking English) were strongly predictive of improved HbA1c over time, explaining roughly half the variability in our patient population. The fact that older patients had the most improvement suggests that the later onset phenotype of type 2 diabetes may be more responsive to therapy. These results complement a recent study that found among T2DM patients with Hba1c < 7.0%, older patients were less likely to have worsening glycemic control over the next year than younger patients [18]. Taken together, these results suggest that more aggressive treatment approaches may be needed for younger patients to achieve similar success. It is perhaps not surprising that patients with the highest HbA1c improved the most, since they had the greatest margin for change. These results support therapeutic optimism rather than resignation regarding patients with the poorest control. Finally, the barrier of not speaking English highlights the critical role of effective communication in achieving goals of care over time. It is also possible that speaking English is a proxy for educational level, another factor that may play a role in achieving diabetes control.

A significant proportion of our baseline cohort (26%) was lost to follow-up. In contrast to the loss to follow-up associated with clinical trials, this loss is reflective of the real world setting in which many patients in our increasingly mobile society (particularly younger and poorer patients) do not remain in the same care setting over time. The demographic differences at baseline underscore the fact that these patients were not lost at random. In our structured chart review, we found that patients with poor control who were subsequently lost to follow-up were less likely to have private insurance and more likely to have drug or alcohol problems than similarly poorly controlled patients who remained in care. These results suggest that 0.4% 10-year HbA1c improvement may be an over-estimate, at least among patients with poor baseline control. However, we did see (in Figure 2) similar annual trends of HbA1c improvement among patients ultimately lost to follow-up for the duration of their care in the system after 1996.

The fate of our 1996 cohort also underscores the lethality of diabetes, with over one-third of our patients dying before 2006. Patients who died were demographically distinct from our follow-up cohorts, with significantly older mean age. For the purposes of our analysis, we did not focus further on this group of patients, since our primary goal was to track HbA1c changes over ten-years among patients with a greater than 10-year life expectancy (i.e. the population of patients most likely to benefit from tight glycemic control). Taking the lost to follow-up and mortality cohorts together, nearly two-thirds of patients present in 1996 were not in our system in 2006. Thus, the improvement in HbA1c control we found over the decade of study may not be generalizable to the overall U.S. population.

The results of this study support the findings in adult participants from the National Health and Nutrition Examination Survey (NHANES). The earlier of the two analyses found that glycemic control rates (HbA1c < 7%) declined from 44.5% in NHANES III (1988-1994) to 35.8% in NHANES 1999-2000 [8] despite improvements in treatment and understanding of the disease during this period. These initial findings, however, were reversed in the later study, however, suggesting that a significant improvement in glycemic control occurred between 1999 and 2004 [7]. Our results also reveal a similar magnitude of 10-year decline in HbA1c compared to a smaller study conducted in our system between 1985 and 1993 in which HbA1c's in 137 patients with T2DM declined from 8.8% to 8.4% (P = 0.09) [9]. Overall, these results demonstrate that at the individual (rather than population) level, patients with T2D have had an incremental decline in HbA1c despite increasing diabetes duration over the past two decades.

Our results must be interpreted in the context of the study design. Because we included all eligible patients in 1996 (rather than selecting patients based on specific HbA1c levels), our results are not biased by "regression to the mean" seen in other studies that pre-select patients at one of the extremes of the outcome distribution. However, clinical data for our overall patient cohort were limited to electronic medical record, laboratory, and medical billing claims data. While this approach allowed us to efficiently analyze one of the largest reported 10-year "usual care" clinical cohorts for type 2 diabetes, we could not provide specific information about actual medication prescription patterns. To address the need for more detailed data, we performed two structured chart reviews. The analysis of the patients very poorly controlled at baseline (HbA1c > 11.5%) provided insight into the social barriers facing patients with poor control who were lost to follow-up (i.e. alcohol and drug use, inadequate insurance, and language discordance). The comparison of patients who improved vs. those who worsened over the next decade revealed higher use of insulin in 1996 among patients who worsened, suggesting that insulin use in that earlier era is a strong marker for greater disease severity; conversely, initiation of insulin after 1996 may have been an important factor among those who improved.

## Conclusion

We found that among patients who remained in our health care system, HbA1c levels declined significantly despite ten years of disease progression and patient aging. Improvements in lipid control were even more pronounced and provide corresponding hope that rates of cardiovascular disease may continue to decline in this high-risk population. Older patients with favorable 10-year prognoses were more likely to achieve glycemic improvement than younger patients, suggesting that the current treatment of younger patients requires a greater therapeutic emphasis to achieve comparable long-term glycemic and cholesterol benefits as older patients.

## Abbreviations

(T2D): Type 2 diabetes; (NHANES): National Health and Nutritional Examination Survey; (MGH): Massachusetts General Hospital; (HbA1c): Hemoglobin A1c;

## Competing interests

The authors declare that they have no competing interests.

## Authors' contributions

KLB, MEL, DMN, and RWG made substantial contributions to conception and design, KJB, MEL, and GW to analysis and interpretation of data; KJB and RWG drafted the manuscript and MEL, GW, DMN revised it critically for important intellectual content; and all authors have given final approval of the version to be published.

## Pre-publication history

The pre-publication history for this paper can be accessed here:

http://www.biomedcentral.com/1472-6963/10/158/prepub
